# Supplementary material for: Risk of Subsequent Primary Cancer in Thyroid Cancer Survivors: A Nationwide Population-Based Study
Source: Diagnostics (Basel). 2023 Sep 11;13(18):2903. doi: 10.3390/diagnostics13182903 (PMC10527961; doi:10.3390/diagnostics13182903)
Supplement: Supplementary file 1 [file diagnostics-13-02903-s001.zip › diagnostics-2577169-supplementary.pdf]

Supplementary table S1. Baseline characteristics of study participants with health screening data

	Overall	Case	Control	*P-value*
Total number, n	258,585	141,952	116,633	
Age (years), mean ± SD	49.9 ± 10.9	49.8 ± 10.8	50.0 ± 11.0	< 0.001
Sex, n (%)				< 0.001
Male	60,133 (23.3)	32,490 (22.9)	27,643 (23.7)	
Female	198,452 (76.8)	109,462 (77.1)	88,990 (76.3)	
Region of residence, n (%)				< 0.001
Urban	126,209 (48.8)	69,979 (49.3)	56,230 (48.2)	
Rural	132,376 (51.2)	71,973 (50.7)	60,403 (51.8)	
Income level, n (%)				< 0.001
1 (lowest)	41,140 (15.9)	22,268 (15.7)	18,872 (16.2)	
2	37,871 (14.6)	20,661 (14.6)	17,210 (14.8)	
3	42,088 (16.3)	23,618 (16.6)	18,470 (15.8)	
4	56,368 (21.8)	31,448 (22.2)	24,920 (21.4)	
5 (highest)	81,118 (31.4)	43,957 (31.0)	37,161 (31.9)	
CCI score				<0.001
0	63,422 (24.5)	63,340 (44.6)	82 (0.1)	
1	88,756 (34.3)	39,467 (27.8)	49,289 (42.3)	
2	34,359 (13.3)	18,985 (13.4)	15,374 (13.2)	
3	22,875 (8.8)	13,174 (9.3)	9,701 (8.3)	
≥4	16,767 (6.5)	10,055 (7.1)	6,712 (5.8)	
Obesity[a]				<0.001
Underweight	7,324 (2.8)	3,472 (2.4)	3,852 (3.3)	
Normal	100,905 (39.0)	52,170 (36.8)	48,735 (41.8)	
Overweight	63,497 (24.6)	34,679 (24.4)	28,818 (24.7)	
Obesity I	75,443 (29.2)	44,316 (31.2)	31,127 (26.7)	
Obesity II	11,416 (4.4)	7,315 (5.2)	4,101 (3.5)	
Smoking				<0.001
Never	211,094 (81.6)	116,839 (82.3)	94,255 (80.8)	
Former	22,508 (8.7)	13,408 (9.4)	9,100 (7.8)	
Current	24,983 (9.7)	11,705 (8.2)	13,278 (11.4)	

CCI, Charlson Comorbidity Index. [a]Body mass index (kg/m^2) was categorized as <18.5 (underweight), ≥18.5 to <23 (normal), ≥23 to <25 (overweight), ≥25 to <30 (obesity I), and ≥30 (obesity II).

Supplementary table S2. Incidence and hazard ratios of subsequent primary cancer for patients with thyroid cancer and matched controls (participants with data on obesity and smoking status).

Type of SPC	Case (n = 141,952)	Control (n = 116,633)	Crude HR (95% CI)	Adjusted HR[a] (95% CI)
All SPCs	25,660 (18.1)	14,797 (12.7)	1.67 (1.64-1.71)	1.58 (1.55-1.62)
Head and Neck (C00–14, C30–32)	532 (0.4)	243 (0.2)	2.07 (1.78-2.41)	1.92 (1.64-2.24)
Digestive system (C15–26)	10,811 (7.6)	6,662 (5.7)	1.57 (1.52-1.62)	1.47 (1.42-1.51)
Gastrointestinal tract (C15–20)	4,721 (3.3)	2,709 (2.3)	1.67 (1.59-1.75)	1.60 (1.52-1.68)
Esophagus (C15)	74 (0.1)	29 (0.0)	2.50 (1.62-3.85)	2.35 (1.51-3.63)
Stomach (C16)	1,377 (1.0)	1,008 (0.9)	1.32 (1.22-1.44)	1.27 (1.17-1.38)
Colon and rectum (C18–20)	3,226 (2.3)	1,642 (1.4)	1.86 (1.76-1.98)	1.79 (1.68-1.90)
Hepato-biliary-pancreatic (C22–25)	5,814 (4.1)	3,728 (3.2)	1.52 (1.46-1.58)	1.39 (1.34-1.45)
Liver and biliary tract (C22–24)	4,041 (2.8)	2,520 (2.2)	1.55 (1.47-1.63)	1.42 (1.35-1.49)
Pancreas (C25)	1,773 (1.2)	1,208 (1.0)	1.45 (1.35-1.57)	1.33 (1.24-1.44)
Lung (C34)	2,172 (1.5)	1,149 (1.0)	1.85 (1.72-1.99)	1.79 (1.66-1.92)
Bone and soft tissue (C40–C41)	121 (0.1)	48 (0.0)	2.32 (1.66-3.25)	2.27 (1.62-3.19)
Limb (C40)	41 (0.0)	10 (0.0)	3.73 (1.86-7.46)	3.50 (1.74-7.05)
Other sites (C41)	80 (0.1)	38 (0.0)	1.95 (1.32-2.88)	1.94 (1.31-2.88)
Skin (C43–44)	343 (0.2)	218 (0.2)	1.58 (1.33-1.87)	1.52 (1.28-1.81)
Breast (C50)	3,091 (2.2)	1,352 (1.2)	2.08 (1.96-2.22)	2.02 (1.90-2.16)
Female genital system (C51–58)	3,332 (2.3)	2,136 (1.8)	1.46 (1.38-1.54)	1.40 (1.33-1.48)
Cervix (C53)	581 (0.4)	395 (0.3)	1.33 (1.17-1.52)	1.27 (1.12-1.45)
Uterus (C54–C55)	842 (0.6)	581 (0.5)	1.42 (1.28-1.58)	1.35 (1.21-1.51)
Ovary (C56–C57)	1,892 (1.3)	1,149 (1.0)	1.53 (1.42-1.64)	1.48 (1.37-1.59)
Male genital system (C60–C63)	1,788 (1.3)	1,382 (1.2)	1.51 (1.41-1.62)	1.38 (1.29-1.49)
Prostate (C61)	1,752 (1.2)	1,359 (1.2)	1.50 (1.40-1.62)	1.38 (1.28-1.48)
Urinary system (C64–C68)	1,430 (1.0)	974 (0.8)	1.46 (1.35-1.59)	1.37 (1.26-1.49)
Kidney (C64)	560 (0.4)	300 (0.3)	1.84 (1.60-2.12)	1.68 (1.46-1.94)
Bladder (C67)	757 (0.6)	621 (0.5)	1.27 (1.14-1.41)	1.21 (1.08-1.34)
Eye, orbit (C69)	34 (0.0)	16 (0.0)	2.03 (1.12-3.70)	2.09 (1.14-3.84)
Brain, CNS (C70–C72)	274 (0.2)	121 (0.)	2.19 (1.77-2.72)	2.08 (1.68-2.59)
Adrenal gland (C74)	77 (0.1)	20 (0.0)	3.56 (2.17-5.82)	3.42 (2.08-5.63)
Hematologic system (C81–C96)	755 (0.5)	319 (0.3)	2.28 (1.99-2.60)	2.10 (1.84-2.40)
Hodgkin Disease (C81)	11 (0.0)	7 (0.0)	1.50 (0.58-3.90)	1.21 (0.46-3.19)
Non-Hodgkin lymphoma (C82–C86)	317 (0.2)	111 (0.1)	2.66 (2.14-3.30)	2.41 (1.93-3.00)
Multiple myeloma (C90)	166 (0.1)	95 (0.1)	1.75 (1.35-2.25)	1.59 (1.23-2.05)
Leukemia (C91–C95)	183 (0.1)	64 (0.1)	2.77 (2.08-3.69)	2.63 (1.97-3.52)

SPC, subsequent primary cancer; HR, hazard ratio; CI, confidence interval; C, diagnostic code used in the International Classification of Diseases (ICD)-10; CNS, central nervous system. Hazard ratios were determined by Cox proportional hazard models. [a]Adjusted HR for age, sex, region of residence, income, Charlson Comorbidity Index, obesity, and smoking status.

Supplementary table S3. Incidence and hazard ratios of subsequent primary cancer for patients with thyroid cancer and matched controls adjusted according to sex.

Type of SPC	Male			Female		
	Case (n = 82,470)	Control (n = 82,469)	Adjusted HR (95% CI)[a]	Case (n = 350,184)	Control (n = 350,185)	Adjusted HR (95% CI)[a]
All SPCs	16,614 (20.2)	11,410 (13.8)	1.51 (1.47-1.55)	61,970 (17.7)	38,569 (11.0)	1.66 (1.63-1.68)
Head and Neck (C00–14, C30–32)	771 (0.9)	239 (0.3)	2.81 (2.43-3.26)	1,299 (0.4)	614 (0.2)	2.05 (1.86-2.26)
Digestive system (C15–26)	6,802 (8.3)	5,179 (6.3)	1.36 (1.31-1.41)	24,716 (7.1)	16,887 (4.8)	1.45 (1.42-1.48)
Gastrointestinal tract (C15–20)	3,372 (4.1)	2,311 (2.8)	1.46 (1.38-1.54)	10,832 (3.1)	6,578 (1.9)	1.61 (1.56-1.67)
Esophagus (C15)	152 (0.2)	49 (0.1)	2.70 (1.94-3.75)	131 (0.0)	54 (0.0)	2.37 (1.71-3.27)
Stomach (C16)	1,149 (1.4)	991(1.2)	1.15 (1.06-1.26)	2,946 (0.8)	2,267 (0.7)	1.26 (1.19-1.33)
Colon and rectum (C18–20)	2,033 (2.5)	1,245 (1.5)	1.65 (1.53-1.78)	7,653 (2.2)	4,184 (1.2)	1.80 (1.74-1.87)
Hepato-biliary-pancreatic (C22–25)	3,327 (4.0)	2,743 (3.3)	1.30 (1.24-1.37)	13,221 (3.8)	9,689 (2.8)	1.37 (1.33-1.40)
Liver and biliary tract (C22–24)	2,516 (3.1)	2,049 (2.5)	1.31 (1.24-1.39)	8,967 (2.6)	6,569 (1.9)	1.37 (1.32-1.41)
Pancreas (C25)	811 (1.0)	694 (0.8)	1.27 (1.14-1.41)	4,254 (1.2)	3,120 (0.9)	1.36 (1.30-1.43)
Lung (C34)	1,482 (1.8)	925 (1.1)	1.71 (1.57-1.86)	5,131 (1.5)	2,757 (0.8)	1.86 (1.77-1.95)
Bone and soft tissue (C40–C41)	121 (0.2)	24 (0.0)	4.92 (3.15-7.69)	299 (0.1)	114 (0.0)	2.55 (2.04-3.17)
Limb (C40)	29 (0.0)	2 (0.0)	15.56 (3.67-65.98)	89 (0.0)	34 (0.0)	2.62 (1.75-3.92)
Other sites (C41)	92 (0.1)	22 (0.0)	3.99 (2.48-6.40)	210 (0.1)	80 (0.0)	2.51 (1.94-3.27)
Skin (C43–44)	221 (0.3)	163 (0.2)	1.43 (1.16-1.76)	875 (0.3)	568 (0.2)	1.60 (1.43-1.78)
Breast (C50)	76 (0.1)	7 (0.0)	10.48 (4.78-22.95)	10,382 (3.0)	5,166 (1.5)	2.12 (2.05-2.20)
Female genital system (C51–58)	0 (0.0)	0 (0.0)	-	10,810 (3.1)	8,312 (2.4)	1.47 (1.43-1.51)
Cervix (C53)	0 (0.0)	0 (0.0)	-	1,975 (0.6)	1,518 (0.4)	1.35 (1.26-1.45)
Uterus (C54–C55)	0 (0.0)	0 (0.0)	-	2,689 (0.8)	2,123 (0.6)	1.46 (1.37-1.54)
Ovary (C56–C57)	0 (0.0)	0 (0.0)	-	6,090 (1.7)	4,615 (1.3)	1.52 (1.46-1.58)
Male genital system (C60–C63)	4,549 (5.5)	3,815 (4.6)	1.25 (1.20-1.31)	0 (0.0)	0 (0.0)	-
Prostate (C61)	4,478 (5.4)	3,764 (4.6)	1.25 (1.19-1.31)	0 (0.0)	0 (0.0)	-
Urinary system (C64–C68)	1,053 (1.3)	604 (0.7)	1.78 (1.60-1.97)	3,124 (0.9)	2,336 (0.7)	1.38 (1.30-1.46)
Kidney (C64)	513 (0.6)	189 (0.2)	2.68 (2.26-3.18)	1,120 (0.3)	632 (0.2)	1.76 (1.59-1.94)
Bladder (C67)	482 (0.6)	370 (0.5)	1.37 (1.19-1.57)	1,810 (0.5)	1,570 (0.5)	1.21 (1.13-1.30)
Eye, orbit (C69)	17 (0.0)	6 (0.0)	3.35 (1.28-8.76)	56 (0.0)	35 (0.0)	1.73 (1.12-2.67)
Brain, CNS (C70–C72)	182 (0.2)	73 (0.1)	2.85 (2.15-3.77)	786 (0.2)	395 (0.1)	2.03 (1.79-2.29)
Adrenal gland (C74)	54 (0.1)	8 (0.01)	6.58 (3.09-14.00)	224 (0.1)	60 (0.0)	3.71 (2.78-4.96)
Hematologic system (C81–C96)	619 (0.8)	252 (0.3)	2.42 (2.08-2.82)	1,860 (0.5)	883 (0.3)	2.07 (1.91-2.25)
Hodgkin Disease (C81)	17 (0.0)	6 (0.0)	2.81 (1.08-7.31)	37 (0.0)	14 (0.0)	2.78 (1.49-5.20)
Non-Hodgkin lymphoma (C82–C86)	301 (0.4)	92 (0.1)	3.06 (2.41-3.88)	864 (0.3)	348 (0.1)	2.29 (2.02-2.60)
Multiple myeloma (C90)	114 (0.1)	76 (0.1)	1.56 (1.16-2.11)	380 (0.1)	264 (0.1)	1.46 (1.24-1.71)
Leukemia (C91–C95)	133 (0.2)	45 (0.1)	2.90 (2.05-4.10)	414 (0.1)	164 (0.1)	2.67 (2.22-3.21)

HR, hazard ratio; CI, confidence interval; C, diagnostic code used in the International Classification of Diseases (ICD)-10; CNS, central nervous system. [a]Adjusted HR for Charlson Comorbidity Index.

Supplementary table S4. Incidence and hazard ratios of subsequent primary cancer for patients with thyroid cancer and matched controls adjusted according to age.

Type of SPC	Age < 40 years			Age ≥ 40 years		
	Case (n = 101,950)	Control (n = 101,959)	Adjusted HR (95% CI)[a]	Case (n = 330,704)	Control (n = 330,695)	Adjusted HR (95% CI)[a]
All SPCs	11,660 (11.4)	7,737 (7.6)	1.95 (1.90-2.01)	66,924 (20.2)	42,242 (12.7)	1.59 (1.57-1.61)
Head and Neck (C00–14, C30–32)	218 (0.2)	94 (0.1)	2.62 (2.04-3.36)	1,852 (0.6)	759 (0.2)	2.28 (2.09-2.48)
Digestive system (C15–26)	3,931 (3.9)	2,647 (2.6)	1.84 (1.75-1.94)	27,587 (8.3)	19,419 (5.9)	1.40 (1.37-1.42)
Gastrointestinal tract (C15–20)	1,640 (1.6)	820 (0.8)	2.46 (2.26-2.68)	12,564 (3.8)	8,069 (2.4)	1.51 (1.47-1.56)
Esophagus (C15)	9 (0.0)	5 (0.0)	2.03 (0.65-6.31)	274 (0.1)	98 (0.0)	2.61 (2.06-3.30)
Stomach (C16)	375 (0.4)	301 (0.23)	1.60 (1.37-1.87)	3,720 (1.3)	2,957 (0.9)	1.21 (1.15-1.27)
Colon and rectum (C18–20)	1,245 (1.2)	503 (0.5)	2.99 (2.69-3.33)	8,441 (2.6)	4,926 (1.5)	1.67 (1.62-1.74)
Hepato-biliary-pancreatic (C22–25)	2,186 (2.1)	1,740 (1.7)	1.56 (1.46-1.67)	14,362 (4.3)	10,692 (3.2)	1.33 (1.30-1.36)
Liver and biliary tract (C22–24)	1,634 (1.6)	1,284 (1.3)	1.57 (1.45-1.69)	9,849 (3.0)	7,334 (2.2)	1.33 (1.29-1.37)
Pancreas (C25)	552 (0.5)	456 (0.5)	1.56 (1.37-1.77)	4,513 (1.4)	3,358 (1.0)	1.33 (1.27-1.39)
Lung (C34)	578 (0.6)	312 (0.3)	2.34 (2.03-2.69)	6,035 (1.8)	3,370 (1.0)	1.79 (1.72-1.87)
Bone and soft tissue (C40–C41)	67 (0.1)	17 (0.0)	4.38 (2.54-7.55)	353 (0.1)	121 (0.0)	2.80 (2.27-3.45)
Limb (C40)	27 (0.0)	3 (0.0)	9.48 (2.79-32.14)	91 (0.0)	33 (0.0)	2.79 (1.86-4.18)
Other sites (C41)	40 (0.0)	14 (0.0)	3.32 (1.78-6.18)	262 (0.1)	88 (0.0)	2.80 (2.19-3.58)
Skin (C43–44)	114 (0.1)	68 (0.1)	2.21 (1.62-3.01)	982 (0.3)	663 (0.2)	1.52 (1.37-1.68)
Breast (C50)	1,842 (1.8)	1,071 (1.1)	2.36 (2.19-2.55)	8,616 (2.6)	4,102 (1.2)	2.16 (2.08-2.24)
Female genital system (C51–58)	3,043 (3.0)	2,661 (2.6)	1.59 (1.51-1.68)	7,767 (2.4)	5,651 (1.8)	1.47 (1.42-1.53)
Cervix (C53)	397 (0.4)	394 (0.4)	1.26 (1.09-1.46)	1,578 (0.5)	1,124 (0.3)	1.42 (1.31-1.53)
Uterus (C54–C55)	726 (0.7)	592 (0.6)	1.89 (1.69-2.12)	1,963 (0.6)	1,531 (0.5)	1.38 (1.29-1.48)
Ovary (C56–C57)	1,911 (1.9)	1,661 (1.6)	1.58 (1.47-1.69)	4,179 (1.3)	2,954 (0.9)	1.55 (1.47-1.62)
Male genital system (C60–C63)	236 (0.2)	192 (0.2)	1.61 (1.33-1.97)	4,313 (1.3)	3,623 (1.1)	1.17 (1.12-1.23)
Prostate (C61)	211 (0.2)	180 (0.2)	1.56 (1.27-1.92)	4,267 (1.3)	3,584 (1.1)	1.17 (1.12-1.23)
Urinary system (C64–C68)	392 (0.4)	327 (0.3)	1.60 (1.37-1.86)	3,785 (1.2)	2,613 (0.8)	1.46 (1.39-1.54)
Kidney (C64)	165 (0.2)	99 (0.1)	2.13 (1.65-2.76)	1,468 (0.4)	722 (0.2)	1.97 (1.80-2.16)
Bladder (C67)	207 (0.2)	210 (0.2)	1.36 (1.11-1.66)	2,085 (0.6)	1,730 (0.5)	1.25 (1.17-1.33)
Eye, orbit (C69)	6 (0.0)	7 (0.0)	1.55 (0.49-4.87)	67 (0.0)	34 (0.0)	2.06 (1.35-3.14)
Brain, CNS (C70–C72)	163 (0.2)	87 (0.1)	2.08 (1.59-2.72)	805 (0.2)	381 (0.1)	2.16 (1.91-2.45)
Adrenal gland (C74)	62 (0.1)	10 (0.0)	6.66 (3.38-13.12)	216 (0.1)	58 (0.0)	3.63 (2.71-4.88)
Hematologic system (C81–C96)	386 (0.4)	160 (0.2)	2.74 (2.27-3.32)	2,093 (0.6)	975 (0.3)	2.07 (1.92-2.24)
Hodgkin Disease (C81)	16 (0.0)	9 (0.0)	1.67 (0.72-3.88)	38 (0.0)	11 (0.0)	3.62 (1.83-7.15)
Non-Hodgkin lymphoma (C82–C86)	180 (0.2)	62 (0.1)	3.04 (2.26-4.08)	985 (0.3)	378 (0.1)	2.39 (2.12-2.69)
Multiple myeloma (C90)	46 (0.1)	27 (0.0)	2.21 (1.34-3.62)	448 (0.1)	313 (0.1)	1.43 (1.23-1.66)
Leukemia (C91–C95)	114 (0.1)	36 (0.0)	3.69 (2.51-5.42)	433 (0.1)	173 (0.1)	2.53 (2.11-3.03)

HR, hazard ratio; CI, confidence interval; C, diagnostic code used in the International Classification of Diseases (ICD)-10; CNS, central nervous system. [a]Adjusted HR for Charlson Comorbidity Index.

Supplementary table S5-1. Incidence and hazard ratios of subsequent primary cancer for patients with thyroid cancer and matched controls adjusted according to latency period (continued)

Type of SPC	<2.0 years			2.0-4.9 years		
	Case (n = 81,673)	Control (n = 68,701)	Adjusted HR (95% CI)[a]	Case (n = 84,243)	Control (n = 89,264)	Adjusted HR (95% CI)[a]
All SPCs	34,870 (42.7)	13,450 (19.6)	3.12 (3.05-3.18)	19,373 (23.0)	12,629 (14.2)	2.21 (2.16-2.26)
Head and Neck (C00–14, C30–32)	1,368 (1.7)	331 (0.5)	4.47 (3.96-5.06)	369 (0.4)	266 (0.3)	1.87 (1.59-2.20)
Digestive system (C15–26)	13,419 (16.4)	6,035 (8.8)	2.68 (2.60-2.77)	8,152 (9.7)	5,613 (6.3)	2.05 (1.98-2.12)
Gastrointestinal tract (C15–20)	7,742 (9.5)	2,740 (4.0)	3.15 (3.00 -3.30)	2,978 (3.5)	2,212 (2.5)	1.90 (1.79-2.01)
Esophagus (C15)	186 (0.2)	22 (0.0)	9.24 (5.88-14.52)	53 (0.1)	28 (0.0)	2.57 (1.61-4.11)
Stomach (C16)	1,969 (2.4)	1,079 (1.6)	2.03 (1.88-2.19)	961 (1.1)	822 (0.9)	1.66 (1.51-1.83)
Colon and rectum (C18–20)	5,515 (6.8)	1,608 (2.3)	3.84 (3.62-4.06)	1,926 (2.3)	1,336 (1.5)	2.03 (1.89-2.18)
Hepato-biliary-pancreatic (C22–25)	5,409 (6.6)	3,131 (4.6)	2.28 (2.18-2.38)	4,974 (5.9)	3,191 (3.6)	2.20 (2.10-2.31)
Liver and biliary tract (C22–24)	4,063 (5.0)	2,312 (3.4)	2.33 (2.21-2.46)	3,481 (4.1)	2,284 (2.6)	2.16 (2.05-2.28)
Pancreas (C25)	1,346 (1.7)	819 (1.2)	2.13 (1.94-2.33)	1,493 (1.8)	907 (1.0)	2.31 (2.12-2.51)
Lung (C34)	2,732 (3.4)	895 (1.3)	3.73 (3.45-4.03)	1,685 (2.0)	925 (1.0)	2.66 (2.45-2.89)
Bone and soft tissue (C40–C41)	242 (0.3)	49 (0.1)	5.76 (4.20-7.90)	88 (0.1)	34 (0.0)	3.64 (2.43-5.46)
Limb (C40)	66 (0.1)	14 (0.0)	5.71 (3.14-10.38)	21 (0.0)	7 (0.0)	4.41 (1.86-10.45)
Other sites (C41)	176 (0.2)	35 (0.1)	5.78 (3.99-8.39)	67 (0.1)	27 (0.0)	3.46 (2.19-5.47)
Skin (C43–44)	397 (0.5)	167 (0.2)	2.95 (2.45-3.56)	287 (0.3)	173 (0.2)	2.41 (1.98-2.92)
Breast (C50)	5,702 (7.0)	1,561 (2.3)	4.08 (3.85-4.32)	2,140 (2.5)	1,229 (1.4)	2.71 (2.52-2.92)
Female genital system (C51–58)	3,893 (4.8)	2,099 (3.1)	2.44 (2.31-2.57)	2,896 (3.4)	2,140 (2.4)	2.04 (1.93-2.16)
Cervix (C53)	1,014 (1.2)	531 (0.8)	2.24 (2.01-2.49)	452 (0.5)	406 (0.5)	1.64 (1.43-1.88)
Uterus (C54–C55)	832 (1.0)	469 (0.7)	2.22 (1.98-2.50)	649 (0.8)	503 (0.6)	1.94 (1.72-2.18)
Ovary (C56–C57)	2,019 (2.5)	1,082 (1.6)	2.65 (2.45-2.86)	1,784 (2.1)	1,212 (1.4)	2.24 (2.08-2.42)
Male genital system (C60–C63)	1,779 (2.2)	972 (1.4)	2.31 (2.13-2.51)	1,288 (1.5)	977 (1.1)	1.78 (1.63-1.94)
Prostate (C61)	1,748 (2.1)	958 (1.4)	2.31 (2.13-2.51)	1,271 (1.5)	964 (1.1)	1.78 (1.63-1.94)
Urinary system (C64–C68)	1,451 (1.8)	664 (1.0)	2.63 (2.39-2.89)	1,052 (1.3)	741 (0.8)	2.02 (1.83-2.22)
Kidney (C64)	747 (0.9)	230 (0.3)	3.74 (3.21-4.35)	357 (0.4)	209 (0.2)	2.48 (2.08-2.95)
Bladder (C67)	606 (0.7)	387 (0.6)	1.98 (1.74-2.26)	650 (0.8)	495 (0.6)	1.85 (1.64-2.08)
Eye, orbit (C69)	34 (0.0)	8 (0.0)	5.10 (2.31-11.24)	15 (0.0)	11 (0.0)	2.39 (1.07-5.34)
Brain, CNS (C70–C72)	440 (0.5)	119 (0.2)	4.56 (3.70-5.61)	243 (0.3)	124 (0.1)	2.82 (2.26-3.52)
Adrenal gland (C74)	135 (0.2)	25 (0.0)	6.12 (3.96-9.46)	70 (0.1)	13 (0.0)	8.53 (4.65-15.64)
Hematologic system (C81–C96)	1,318 (1.6)	363 (0.5)	3.85 (3.42-4.34)	479 (0.6)	239 (0.3)	2.90 (2.47-3.40)
Hodgkin Disease (C81)	35 (0.0)	9 (0.0)	4.21 (1.99-8.9)	12 (0.0)	5 (0.0)	3.14 (1.09-9.09)
Non-Hodgkin lymphoma (C82–C86)	819 (1.0)	184 (0.3)	4.38 (3.72-5.16)	156 (0.2)	91 (0.1)	2.45 (1.88-3.19)
Multiple myeloma (C90)	156 (0.2)	59 (0.1)	3.26 (2.39-4.45)	104 (0.1)	80 (0.1)	1.76 (1.31-2.37)
Leukemia (C91–C95)	191(0.2)	76 (0.1)	2.89 (2.19-3.80)	160 (0.2)	37 (0.0)	6.61 (4.59-9.53)

Supplementary table S5-2. Incidence and hazard ratios of subsequent primary cancer for patients with thyroid cancer and matched controls adjusted according to latency period.

Type of SPC	5.0-9.9 years			≥10.0 years		
	Case (n = 170,240)	Control (n = 134,662)	Adjusted HR (95% CI)[a]	Case (n = 96,498)	Control (n = 140,027)	Adjusted HR (95% CI)[a]
All SPCs	18,939 (11.1)	15,513 (11.5)	1.12 (1.10-1.15)	5,402 (5.6)	8,387 (6.0)	1.21 (1.16-1.25)
Head and Neck (C00–14, C30–32)	261 (0.2)	175 (0.1)	1.34 (1.10-1.63)	72 (0.1)	81 (0.1)	1.59 (1.15-2.21)
Digestive system (C15–26)	7,854 (4.6)	6,888 (5.1)	1.02 (0.98-1.05)	2,093 (2.2)	3,530 (2.5)	1.07 (1.01-1.13)
Gastrointestinal tract (C15–20)	2,760 (1.6)	2,636 (2.0)	0.93 (0.88-0.99)	724 (0.8)	1,301 (0.9)	1.01 (0.92-1.11)
Esophagus (C15)	33 (0.0)	30 (0.0)	0.91 (0.55-1.50)	11 (0.0)	23 (0.0)	0.79 (0.38-1.65)
Stomach (C16)	930 (0.6)	908 (0.7)	0.93 (0.85-1.02)	235 (0.2)	449 (0.3)	0.94 (0.80-1.11)
Colon and rectum (C18–20)	1,774 (1.0)	1,673 (1.2)	0.94 (0.88-1.01)	471 (0.5)	812 (0.6)	1.06 (0.94-1.19)
Hepato-biliary-pancreatic (C22–25)	4,857 (2.9)	4,016 (3.0)	1.08 (1.03-1.13)	1,308 (1.4)	2,094 (1.4)	1.13 (1.05-1.21)
Liver and biliary tract (C22–24)	3,175 (1.9)	2,727 (2.0)	1.05 (0.99-1.11)	764 (0.8)	1,295 (0.9)	1.09 (0.99-1.20)
Pancreas (C25)	1,682 (1.0)	1,289 (1.0)	1.15 (1.06-1.24)	544 (0.6)	799 (0.6)	1.19 (1.06-1.33)
Lung (C34)	1,691 (1.0)	1,176 (0.9)	1.30 (1.20-1.40)	505 (0.5)	686 (0.5)	1.28 (1.13-1.44)
Bone and soft tissue (C40–C41)	74 (0.0)	41(0.0)	1.59 (1.08-2.35)	16 (0.0)	14 (0.0)	2.21 (1.05-4.65)
Limb (C40)	26 (0.0)	10 (0.0)	2.26 (1.07-4.77)	5 (0.0)	5 (0.0)	2.56 (0.71-9.29)
Other sites (C41)	48 (0.0)	31 (0.0)	1.37 (0.87-2.18)	11 (0.0)	9 (0.0)	2.04 (0.82-5.08)
Skin (C43–44)	320 (0.2)	251 (0.2)	1.15 (0.97-1.36)	92 (0.1)	140 (0.1)	1.17 (0.89-1.53)
Breast (C50)	2,039 (1.2)	1,537 (1.1)	1.31 (1.23-1.41)	577 (0.6)	846 (0.6)	1.42 (1.27-1.59)
Female genital system (C51–58)	3,034 (1.8)	2,556 (1.9)	1.18 (1.12-1.24)	987 (1.0)	1,517 (1.1)	1.36 (1.26-1.48)
Cervix (C53)	386 (0.2)	393 (0.3)	0.95 (0.82-1.10)	123 (0.1)	188 (0.1)	1.35 (1.07-1.71)
Uterus (C54–C55)	875 (0.5)	650 (0.5)	1.35 (1.22-1.50)	333 (0.3)	501 (0.4)	1.37 (1.19-1.59)
Ovary (C56–C57)	1,761 (1.0)	1,497 (1.1)	1.17 (1.09-1.26)	526 (0.5)	824 (0.6)	1.36 (1.21-1.52)
Male genital system (C60–C63)	1,179 (0.7)	1,237 (0.9)	0.84 (0.78-0.91)	303 (0.3)	629 (0.4)	0.85 (0.74-0.98)
Prostate (C61)	1,159 (0.7)	1,223 (0.9)	0.84 (0.77-0.91)	300 (0.3)	619 (0.4)	0.86 (0.74-0.99)
Urinary system (C64–C68)	1,257 (0.7)	974 (0.7)	1.18 (1.08-1.28)	417 (0.4)	561 (0.4)	1.34 (1.18-1.53)
Kidney (C64)	426 (0.3)	256 (0.2)	1.52 (1.30-1.79)	103 (0.1)	126 (0.1)	1.53 (1.17-2.01)
Bladder (C67)	746 (0.4)	663 (0.5)	1.02 (0.92-1.14)	290 (0.3)	395 (0.3)	1.31 (1.12-1.53)
Eye, orbit (C69)	20 (0.0)	11 (0.0)	1.83 (0.85-3.94)	4 (0.0)	11 (0.0)	0.81 (0.25-2.63)
Brain, CNS (C70–C72)	216 (0.1)	144 (0.1)	1.44 (1.16-1.79)	69 (0.1)	81 (0.1)	1.49 (1.07-2.07)
Adrenal gland (C74)	63 (0.0)	17 (0.0)	3.54 (2.05-6.13)	10 (0.0)	13 (0.0)	1.40 (0.60-3.29)
Hematologic system (C81–C96)	528 (0.3)	337 (0.3)	1.42 (1.24-1.64)	154 (0.2)	196 (0.1)	1.38 (1.11-1.72)
Hodgkin Disease (C81)	4 (0.0)	2 (0.00)	1.75 (0.31-9.76)	3 (0.0)	4 (0.0)	1.58 (0.34-7.40)
Non-Hodgkin lymphoma (C82–C86)	147 (0.1)	119 (0.1)	1.11 (0.87-1.42)	43 (0.0)	46 (0.0)	1.75 (1.13-2.69)
Multiple myeloma (C90)	174 (0.1)	112 (0.1)	1.33 (1.04-1.69)	60 (0.1)	89 (0.1)	1.11 (0.79-1.55)
Leukemia (C91–C95)	155 (0.1)	65 (0.1)	2.37 (1.76-3.19)	41 (0.0)	31 (0.0)	2.48 (1.53-4.02)

HR, hazard ratio; CI, confidence interval; C, diagnostic code used in the International Classification of Diseases (ICD)-10; CNS, central nervous system. [a]Adjusted HR for Charlson Comorbidity Index
